# KSHV Targeted Therapy: An Update on Inhibitors of Viral Lytic Replication

**DOI:** 10.3390/v6114731

**Published:** 2014-11-24

**Authors:** Natacha Coen, Sophie Duraffour, Robert Snoeck, Graciela Andrei

**Affiliations:** Rega Institute for Medical Research, KU Leuven, B-3000 Leuven, Belgium; E-Mails: natacha.coen@rega.kuleuven.be (N.C.); sophie.duraffour@rega.kuleuven.be (S.D.); robert.snoeck@rega.kuleuven.be (R.S.)

**Keywords:** KSHV, antiviral, nucleoside analog, DNA polymerase inhibitors, lytic cycle, ganciclovir, cidofovir, foscarnet

## Abstract

Kaposi’s sarcoma-associated herpesvirus (KSHV) is the causative agent of Kaposi’s sarcoma, primary effusion lymphoma and multicentric Castleman’s disease. Since the discovery of KSHV 20 years ago, there is still no standard treatment and the management of virus-associated malignancies remains toxic and incompletely efficacious. As the majority of tumor cells are latently infected with KSHV, currently marketed antivirals that target the virus lytic cycle have shown inconsistent results in clinic. Nevertheless, lytic replication plays a major role in disease progression and virus dissemination. Case reports and retrospective studies have pointed out the benefit of antiviral therapy in the treatment and prevention of KSHV-associated diseases. As a consequence, potent and selective antivirals are needed. This review focuses on the anti-KSHV activity, mode of action and current status of antiviral drugs targeting KSHV lytic cycle. Among these drugs, different subclasses of viral DNA polymerase inhibitors and compounds that do not target the viral DNA polymerase are being discussed. We also cover molecules that target cellular kinases, as well as the potential of new drug targets and animal models for antiviral testing.

## 1. Introduction

In 1872, Moritz Kaposi described a rare angiosarcoma that manifested mainly as skin lesions in elderly men [1]. More than a century passed between the first description of Kaposi’s sarcoma (KS) and the discovery of its etiologic agent, Kaposi’s sarcoma-associated herpesvirus (KSHV), by Chang and Moore in 1994 [2]. KS is a neoplasm derived from lymphatic endothelial cells infected with KSHV, composed of spindle-shaped cells and inflammatory mononuclear cells [3]. KS is grouped into four epidemiological forms: classic, endemic, iatrogenic and AIDS-related [4]. In addition, KSHV has been associated with two other diseases, primary effusion lymphoma (PEL) and multicentric Castleman’s disease (MCD) [5]. PEL is a B-cell lymphoma that develops in pleural, pericardial or peritoneal cavity, while the B-cell lymphoproliferative disorder MCD is predominantly found in the lymph nodes and is characterized by vascular proliferation in the germinal centers [6,7].

## 2. Management of KSHV-Associated Diseases

There are no standard therapeutic guidelines for the management of KSHV-associated diseases, yet the main therapeutic options are discussed below for each disease. The treatment of choice for patients with KS depends on several parameters, such as the tumor location and variant of KS, rate of progression, distribution of the lesions, severity of the symptoms, and immune competence [8]. Therapeutic approaches for classic KS range from no treatment to surgical excision, local delivery of chemotherapeutic agents (such as bleomycin, vinblastine, vincristine and alitretinoin), and radiotherapy (Table 1) [9]. Management of iatrogenic KS often involves reduction or elimination of immunosuppressive therapy with or without local measures, whereas endemic KS is frequently responsive to systemic chemotherapy [10,11]. 

The current first-line systemic therapy for advanced, progressive acquired immunodeficiency syndrome (AIDS)-KS includes liposomal anthracyclines, such as daunorubicin and doxorubicin [12,13]. An essential component in the management of human immunodeficiency virus (HIV)-associated KS is the control of KS progression with highly active antiretroviral therapy (HAART), which leads to both immune reconstitution and control of HIV viremia [12,14,15]. Additional data from HIV cohorts also suggested that specific components of HAART might impact the incidence and resolution of KS [16]. Several randomized, placebo-controlled trials of high-dose zidovudine for the treatment of HIV demonstrated a 36% reduction in risk of developing KS compared with persons receiving placebo alone [17]. To date, there are no comprehensive studies conducted to evaluate whether HAART is able to inhibit KSHV viral production [18], but it has been shown that zidovudine is a substrate for KSHV thymidine kinase (TK) [19]. 

In addition, recent research has shown that HIV protease inhibitors, e.g., nelfinavir, have anti-angiogenic and anti-tumor properties [20]. Therefore, HAART combinations that contain HIV protease inhibitors may be superior for treatment of KS patients than those without [21]. Moreover, anti-herpetic agents, such as ganciclovir (GCV), were shown to reduce plasma viral load of KSHV and can prevent KS in KSHV-seropositive transplant recipients [9]. Additionally, target-based therapies, such as inhibition of angiogenesis, metalloproteinases, and cytokine signaling, may be an effective strategy to treat patients with KS that progresses despite chemotherapy and/or HAART [22]. 

PEL has usually been treated with chemotherapy (Table 1), but the prognosis is very poor in patients with a median survival of less than six months [23]. Individual case reports documented responses to antiviral therapy (GCV, foscarnet (PFA), intracavity cidofovir (CDV, HPMPC)), the proteosome inhibitor bortezomib, the immunosuppressive agent rapamycin, the monoclonal antibody rituximab (which targets the CD20 protein on the surface of B lymphocytes), and the antitumor antibiotic drug bleomycin [24,25,26]. 

In MCD patients, KSHV induces both human IL-6 and virus-encoded IL-6, and, therefore, treatment with tocilizumab, an anti-human IL-6 receptor antibody, has led to clinical responses in these patients [27]. Recently, siltuximab, a chimeric monoclonal antibody against IL-6, has been developed for the treatment of MCD patients showing promising results in a phase I clinical trial [28]. Rituximab therapy has been evaluated for the treatment of MCD and up to 70% of patients responded to the therapy (Table 1) [24,29,30]. In addition, antiviral therapy with GCV has been reported successful in MCD patients, since this disease is associated with active KSHV replication [31].

**Table 1:** Treatment modalities of KSHV-related diseases.

Treatment		KSHV-related Diseases
**Intensification of HAART**		AIDS-KS
**Surgical excision**		KS (single skin lesion)
**Radiotherapy**		KS
**Immunotherapy**	Reduction of immunosuppressive therapy	KS, PEL and MCD
	Anti-CD20 (Rituximab) Anti-human IL-6 receptor (Tocilizumab) Anti-IL6 chimeric monoclonal antibody (Siltuximab)	MCD
**Chemotherapy**	Liposomal anthracyclines	KS
	CHOP (cyclophosphamide, doxorubicin, vincristine, prednisone)	PEL and MCD
**Antiviral drugs**	(Val)ganciclovir, foscarnet	KS, PEL and MCD
	Intracavity cidofovir	PEL
**Others**	mTOR inhibitor (Rapamycin) Proteasome inhibitor (Bortezomib)	KS, PEL PEL
	Paclitaxel, anti-angiogenic agents, matrix metalloproteinase inhibitors	KS

## 3. Antiviral Therapy for the Treatment and Prevention of KSHV-Related Malignancies

Inhibition of KSHV lytic phase by antiviral drugs has not shown great efficacy for the treatment of KS, primarily due to the small amount of lytic KSHV present in KS tumors [32]. Though, the few cells showing lytic replication are known to play a central role in KS tumorigenesis [33]. However, a greater proportion of infected cells in PEL and MCD express lytic phase genes (up to 25% in MCD), as compared to KS, and, therefore, antiviral agents might be more effective in the treatment of MCD and PEL, than of KS [16,33,34].

The use of anti-herpes drugs in the protection against the development of AIDS-associated KS has been evaluated in a few studies. In 1996, analysis of data from 935 homosexual men with AIDS from the Multicenter AIDS Cohort Study showed that ACV did not appear to reduce the risk of KS [35]. In contrast, among men with cytomegalovirus (CMV) disease, GCV and PFA use were associated (although not significant) with a reduced risk of KS. An observational study has also suggested that GCV and PFA, but not acyclovir (ACV), may prevent the development of KS in HIV-infected patients [35]. Another study performed in the United Kingdom where a total of 3688 HIV patients were followed up for a median period of 4.2 years, during which time 598 patients developed KS, also indicated that GCV and PFA may have some activity in preventing the occurrence of KS, but that ACV had no benefit [36]. In a prospective, randomized, double-blind, placebo-controlled study including CMV-infected persons with advanced AIDS stage, prophylactic oral GCV significantly reduced the risk of CMV disease but not significant differences between the placebo and GCV groups were observed in the 12-month Kaplan-Meir estimates of KS (12% in the placebo group and 8% in the GCV group) [37]. The safety and efficacy of valganciclovir (VGCV, the oral prodrug of GCV) on HHV-8 replication in the oropharynx in HIV-seropositive and HIV-negative persons who were asymptomatically infected with HHV-8 was determined in a randomized, double-blind, placebo-controlled, crossover trial. VGCV administered orally once a day proved to be well-tolerated and significantly reduced the frequency and quantity of KSHV replication [38]. 

Regarding therapy of KS, a pilot study of CDV including seven patients with KS (five patients with AIDS-KS and two with classical KS) did not provide proof of principle for the treatment of KS with CDV [39]. Despite treatment with CDV (5 mg/kg/dose weekly for two weeks and then every other week) all patients had progression of their KS and there was no decrease in the virus load in peripheral blood mononuclear cells. Another report described the effects of CDV in two patients with AIDS-KS that received the same schedule of treatment for a period of 10 and 12 months [40]. An important regression of cutaneous KS lesions was observed after three months of treatment and reactivation of new KS lesions was not observed until six and 15 months after the end of the treatment. Treatment of classical KS with intralesional injections of CDV for five weeks gave no clinical, histological, immunohistological, or virological changes compared with saline-injected lesions [41].

KSHV gene expression was studied in CDV-treated and untreated PEL cells following induction to lytic replication with TPA (12-O-tetradecanoylphorbol-13-acetate) [42]. This study revealed that the expression of genes implicated in the pathogenesis of KS or KS-like tumors, such as vGPCR, vIL-6, viral interferon regulatory factor 1 (vIRF-1), and viral macrophage inflammatory protein II (vMIP-II), was not inhibited after treatment with CDV. This is likely also true for GCV or PFA, since they all block viral DNA replication and inhibiting DNA replication does not prevent expression of early genes implicated in viral pathogenesis. This might offer a rationale for the failure of CDV therapy in KSHV-related diseases. 

Successful treatments of PEL with antiviral agents, alone or with adjunctive chemotherapy, immunotherapy or HAART, have been described to date with both GCV [43,44] and CDV [43,45,46]. However, the data on intravenous administration of CDV for treating PEL are limited and also controversial. Complete remission has been documented in two HIV-positive patients with PEL when CDV was combined with antiretroviral and interferon therapies, while another patient achieved only partial remission and died after six months [45,47]. The authors of this case study postulated that the concentrations of CDV achieved in the pleural fluid were not high enough when the drug was administered intravenously. On the contrary, several studies reported achieving partial or complete remission of PEL in HIV-negative patients with intracavity CDV after conventional chemotherapy failure [46,48]. 

Additionally, several MCD patients have been successfully treated with GCV [31,49] , whereas failures have been reported with CDV [50]. In addition, a pilot study conducted with high dose of zidovudine combined with VGCV in patients with symptomatic MCD demonstrated that 12 out of 14 patients had substantial clinical improvement. However, this study was not randomized and controlled trials would be needed to further evaluate the efficacy of zidovudine/VGCV and compare it with other approaches [51].

The impact of antiviral treatment on KS (*i.e.*, KSHV latently-infected cells) could be potentially improved by using combination therapy of antivirals together with lytic inducing agents (leading to KSHV reactivation) [33]. The approach of inducing lytic replication of γ-herpesviruses malignancies that employ lytic activation of viruses latently infecting tumors represent a novel strategy of anti-neoplastic therapy. This strategy, named lytic induction therapy, has been explored for KSHV, but in contrast to Epstein-Barr virus (EBV), has not yet been validated in clinical trials [52]. Agents that induce lytic replication include histone deacetylase inhibitors (HDAC) such as valproic acid, phorbol esters, calcium ionophores, and NF-kappaB inhibitors [53]. *In vitro*, valproic acid has been shown to significantly induce KSHV lytic cycle in PEL cells, resulting in apoptosis of the tumor cells [54]. However, a pilot clinical trial demonstrated that valproic acid was not able to induce KSHV lytic replication in patients with AIDS-associated KS on HAART [55]. Further researches are focusing on studying more potent lytic inducing agents, such as bortezomib, 5-azacytidine and vorinostat (suberanilohydroxamic acid), as well as on increasing the treatment regimens in patients with KS [26,56,57]. In addition to bortezomib, the non-tumor-promoting phorbol ester prostratin was also shown to induce immediate-early, early and late KSHV gene expression from two lymphoma cell lines *in vitro*, suggesting that both drugs could be used as therapeutic agents for KSHV-associated malignancies [58]. Recently, the combination of bortezomib with the HDAC inhibitor vorinostat was found to potently reactivate KSHV lytic replication and to induce PEL cell death, resulting in significantly prolonged survival of PEL-bearing mice [59]. These findings provide a strong rationale for using proteasome/HDAC inhibitor combination for the therapy of PEL.

## 4. Inhibitors of KSHV Lytic Replication under Investigation

Despite the fact that various antiviral agents inhibit KSHV replication *in vitro*, no drugs are currently licensed for the treatment of KSHV-related diseases. From the target perspective, candidate inhibitors for treatment of KSHV-related infections can be divided in two groups, (i) compounds that act on the viral DNA polymerase and (ii) drugs that do not interact with the viral DNA polymerase. The first group of compounds includes nucleoside analogs, acyclic nucleoside phosphonates (ANPs), pyrophosphate analogs and non-nucleoside inhibitors. Their anti-KSHV activities are summarized in Table 2. The second group of inhibitors targeting viral proteins comprises compounds isolated from plants.

### 4.1. DNA Polymerase Inhibitors: Nucleoside Analogs

**Figure 1:**
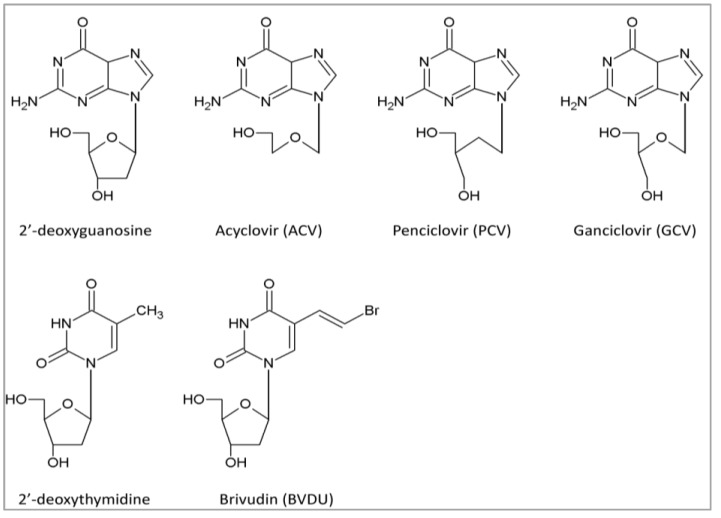
Structures of currently approved nucleoside analogs for herpesvirus infections. Acyclovir (ACV), penciclovir (PCV), ganciclovir (GCV) are derivatives of the natural nucleoside 2’-deoxyguanosine, whereas brivudin (BVDU) is an analog of the natural nucleoside 2’-deoxythymidine.

Nucleoside analogs that are approved for the treatment of herpesvirus infections, other than KSHV and EBV, include ACV, penciclovir (PCV) and GCV and brivudin (BVDU) (Figure 1). 

**Figure 2:**
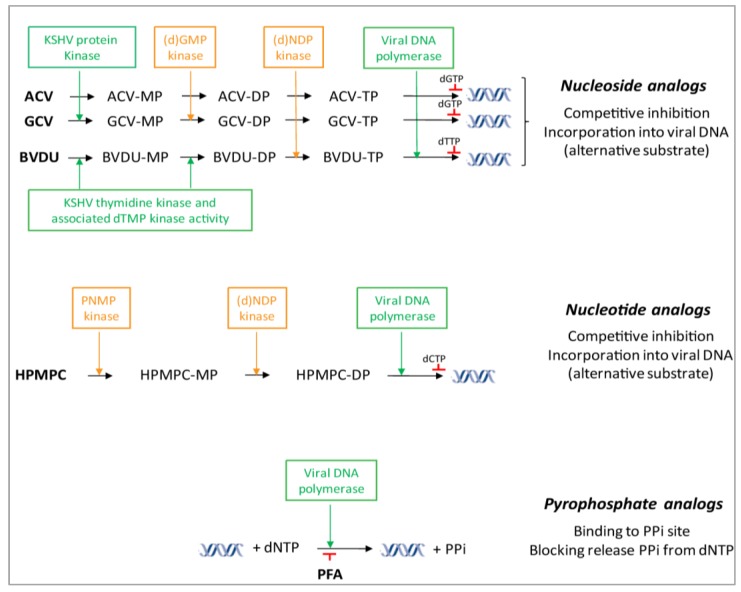
Mechanism of action of viral DNA polymerase inhibitors against KSHV replication. Nucleoside analogs require three phosphorylation steps to become active, being their conversions to the monophosphate (MP) forms carried out by the viral TK (BVDU) or PK (ACV and GCV). Further phosphorylation to the diphosphate (DP) is carried out by the viral TK for BVDU or cellular enzymes for ACV and GCV (*i.e.*, dGMP kinase). Conversion of these drugs to their triphosphate form (TP) by the nucleoside 5’-diphosphate (NDP) kinase results in inhibition of viral DNA polymerases because they act as competitive inhibitors of the natural substrate and/or as alternative substrates if incorporated into the growing DNA chain. ANPs, such as CDV, do not require activation by a virus-encoded enzyme to be active; instead, the two phosphorylations are done by cellular kinases (pyrimidine nucleoside monophosphate (PNMP) and 5’-diphosphate (NDP) kinase). ANP-DPs, recognized by the viral DNA polymerase, will then block DNA synthesis. PFA does not require modifications by viral or cellular kinases. PFA binds to the pyrophosphate (PPi) exchange site of the viral DNA polymerase and blocks the release of pyrophosphate from the terminal nucleoside triphosphate. As a consequence, 3’-5’-phosphodiester linkage necessary for viral DNA elongation is not possible (adapted from [62]).

Nucleoside analogs in their active forms target and inhibit viral DNA polymerases by acting as competitive inhibitors of the natural dNTP substrates and/or by incorporation into the growing DNA chain where they can terminate DNA elongation. To become active, they require three intracellular phosphorylation steps to convert the nucleoside analogs into mono- (MP), di- (DP), and triphosphate (TP) forms (Figure 2). The first phosphorylation step is carried out by viral kinases, limiting this step to virus-infected cells [60], whereas the two subsequent phosphorylations are performed by cellular kinases [deoxyguanosine monophosphate (dGMP) and deoxynucleoside diphosphate (dNDP)] [61]. 

The viral TK of herpes simplex virus type 1 and type 2 (HSV-1 and HSV-2) and varicella-zoster virus (VZV) performs the initial phosphorylation of nucleoside analogs [62]. However, it has been demonstrated that KSHV TK has narrow substrate specificity since it recognizes pyrimidine derivatives (*i.e.*, BVDU) and not purine derivatives (*i.e.*, ACV and GCV) [63]. However, there is still a debate whether purine analogs could be phosphorylated by the KSHV TK to some degree [64]. Purine analogs are activated by the virus protein kinase (PK, ORF36) in KSHV-infected cells. KSHV PK is the homolog of the UL97 protein kinase encoded by human cytomegalovirus (HCMV), which is responsible for the conversion of GCV, and to a lesser extent of ACV, into their monophosphate forms in HCMV-infected cells [65]. 

BVDU is dependent on the virus-encoded TK and on its associated thymidylate kinase (dTMP) activity responsible for the first and second phosphorylations of BVDU and related analogs [66]. Previous studies have shown that KSHV and EBV TK also possesses thymidylate kinase activity [63,67]. The last phosphorylation step in the activation of BVDU is carried out by the cellular (d)NDP kinase.

Once activated, the nucleoside analogs in their triphosphate forms enter in competition with the natural substrates (dGTP or dTTP) for the viral DNA polymerase. They can inhibit the incorporation of natural dGTP or dTTP into the viral DNA in elongation or act as an alternate substrate and be incorporated into the DNA at its 3’-terminus [68]. Once ACV is incorporated, it cannot be excised by the DNA polymerase-associated 3’-5’ exonuclease and it prevents further chain elongation because the 3’-hydroxyl group needed for DNA elongation is missing [69]. It has also been demonstrated that following incorporation of ACV-TP, the viral polymerase becomes trapped on the terminated DNA chain when the next deoxynucleoside triphosphate binds [69]. On the other hand, PCV, GCV and BVDU have a 3’-hydroxyl group on its acyclic side chain, allowing limited chain elongation when incorporated into the growing DNA strand [69,70].

ACV, marketed as Zovirax^®^, represents the first generation of effective anti-herpetic drugs with an excellent safety profile and potent activity against HSV and VZV infections [70]. Due to its limited oral bioavailability, the valine ester of ACV (VACV, Zelitrex^®^ and Valtrex^®^) was developed and proved to be a safe and efficacious prodrug in patients with genital herpes, herpes *labialis*, and herpes zoster [62]. *In vitro*, ACV shows inhibitory activity against EBV, while several studies have consistently reported the weak anti-KSHV properties of ACV (Table 2) [71,72,73,74,75,76]. PCV (Denavir^®^ and Vectavir^®^) and its orally available prodrug, famciclovir (Famvir^®^) are also indicated for the treatment of mucocutaneaous HSV-1 and HSV-2 infections, particularly recurrent herpes *labialis*, and have a spectrum of anti-KSHV activity similar to that of ACV [72,77,78]. Among several purine analogs, an ACV derivative, (1S’,2R’)-9-[[1’,2’-bis(hydroxymethyl)cycloprop-1-yl]methyl]guanine (A-5021) was also shown to lack activity against KSHV, while this drugs was a potent inhibitor of HSV-1, HSV-2, VZV, HHV-6 and EBV replication [79]. Since KSHV does not show great sensitivity to ACV, its derivative H2G [R-9-[4-hydroxy-2-(hydroxymethyl)butyl]guanine] is also not inhibitory for KSHV replication [72]. 

GCV (Cymevene^®^ and Cytovene^®^) was the first antiviral agent approved for the treatment of HCMV infections in immunocompromised patients and remains the first-line treatment of HCMV disease in transplant recipients [62,80]. In addition to HCMV, GCV has demonstrated efficacy against HSV, VZV, EBV, and KSHV replication [72,73,74,76,81]. Several reports have shown the efficacy of GCV against KSHV in the PEL cell line BCBL-1 for which the EC_50_ ranged from 1 µM to 10 µM (Table 2). However, major drawbacks of GCV are its significant bone marrow toxicity, its short half‑life in tissue following oral administration (~5 h) and low bioavailability (~6% for GCV) [82]. Oral bioavailability is significantly improved with its L-valyl-ester derivative, namely VGCV (Valacyte^®^), to approximately 60% [62]. A structural analog of GCV, S2242 [(1,3-dihydroxy-2-propoxymethyl)purine], proved to be a more potent inhibitor of KSHV replication than GCV [72,83]. In contrast to GCV, S2242 is not phosphorylated by a herpesvirus kinase [84,85]. In fact, the cellular deoxyguanosine kinase is responsible for the first phosphorylation step, and subsequently the activation of S2242 [83]. However, the development of S2242 was halted due to toxicity [86]. 

Cyclopropavir, a methylenecyclopropane nucleoside resembling GCV, is under preclinical development for the treatment of HCMV-related infections. It is first phosphorylated by the HCMV PK and, under its active triphosphate form, the drug will further inhibit the viral DNA polymerase [87,88]. This compound has also shown good antiviral activity against KSHV replication *in vitro* for which the EC_50_ was 3.8 µM as measured by DNA hybridization assay [89]. The group of Prichard and colleagues reported that analogs of this class of compounds bearing 6-alkylthio substitutions had inhibitory efficacies comparable to cyclopropavir against KSHV replication *in vitro* (Table 2) [89]. 

The pyrimidine analog, BVDU (Zostex^®^, Brivirac^®^, Zerpex^®^), is a highly selective antiviral agent against HSV-1 and VZV replication and is indicated for the treatment of herpes *labialis* and herpes zoster [70] . BVDU has also shown activity against KSHV, albeit its anti-KSHV antiviral activity may be controversial as it varied from 0.9 µM to 24 µM in BCBL-1 cells depending on the report (Table 2) [72,73,90,91,92]. Still, the *in vitro* antiviral activity of BVDU could not be confirmed *in vivo* against murine γ-herpesvirus (MHV-68) replication in immunocompetent mice [90] and against MHV-68-induced mortality in immunocompromised mice [93]. However, BVDU administered orally at similar concentrations, is highly effective against HSV-1 and VZV infections in terms of reducing virus-induced mortality or diminishing virus titers in infected mice [94,95]. Thus, BVDU does not seem to be a suitable candidate drug for potential treatment of KSHV-related diseases. 

Moreover, 2’-*exo*-methanocarbathymidine [(North)-methanocarbathymidine (N-MCT)], a thymidine analog, was identified as a potent drug with *in vitro* anti-KSHV activity. N-MCT blocked KSHV replication at EC_50_s 5- to 10-fold lower than those of CDV and GCV, without notable cytotoxicity [96]. However, the *in vivo* antiviral efficacy was not investigated. Additionally, despite the higher *in vivo* (MHV-68 mouse model) antiviral efficacy of two thionucleoside derivatives, KAY-2-41 and KAH-39-149, as compared to HDVD, these molecules were active *in vitro* against EBV but not against KSHV [97]. 

While the EC_50_s of zidovudine and stavudine have not been reported, these anti-HIV nucleoside reverse transcriptase inhibitors have been shown to be substrates of γ-herpesvirus TK, which efficiently converts them to their monophosphate forms [19,63]. 

**Table 2:** Anti-KSHV activity of viral DNA polymerase inhibitors.

Class	Subclass	Abbreviation	Drug Name	EC_50_ Range (µM) ^a^	Stage of Development ^d^	Refs.
Nucleoside analogs	Purine analogs	ACV	Acyclovir	26–138	Cohort study	[35,36,72,73,74,75,90]
		PCV	Penciclovir	43	*In vitro*	[72]
		A-5021	(1S,2R)-9-[[1,2-bis(hydroxymethyl) cycloprop-1yl]methyl]guanine	75	*In vitro*	[79]
		H2G	Omaciclovir	42	*In vitro*	[72]
		GCV	Ganciclovir	1.0–10	Randomized, controlled trial (with VGCV)	[31,38,72,73,74,75,90]
		S2242	2-Amino-*7*-[(1,3-dihydroxy-2-propoxy)-methyl]purine	0.1	*In vitro*	[72]
	*Methylenecyclopropane nucleosides*	CPV	Cyclopropavir	3.8 ^b^	*In vitro*	[89]
			6-Alkoxy-substituted derivatives	1.8–3.5 ^b^	*In vitro*	[89]
			6-Alkylthio-substituted derivatives	1.9–7.3 ^b^	*In vitro*	[89]
	Pyrimidine analogs	AZT	Zidovudine		Randomized trial	[17]
		BVDU	Brivudine	0.9–24	*In vivo*	[72,73,90]
		N-MCT	(North)-methanocarbathymidine	0.08	*In vitro*	[96]
	*L-dioxolane uracil analog*	HDVD	1-[(2S,4S-2-(hydroxymethyl)-1,3-dioxolan-4-yl]5-vinylpyrimidine-2,4(1H,3H)-dione	0.09	*In vivo*	[90]
	*Thiothymidine analogs*	KAY-2-41	1-methyl substituted 4-thiothymidine	≥130	*In vivo*	[97]
		KAH-39-149	4-azido substituted 4-thiothymidine	>200	*In vivo*	[97]
Acyclic nucleoside phosphonates	HPMP derivatives	HPMPC, CDV	Cidofovir	0.3–6.3	Pilot study	[39,46,72,73,74,75,90]
		CMX001	Brincidofovir	0.7	*In vitro*	[98]
		HPMP-5-azaC	1-(*S*)-[3-hydroxy-2-(phosphonomethoxy)-propyl]-5-azacytosine	0.7	*In vivo*	[98]
		HPMPA	(*S*)-9-[3-hydroxy-2-(phosphonomethoxy)-propyl]adenine	0.7	*In vitro*	[72,98]
		HPMPDAP	(*S*)-9-[3-hydroxy-2-(phosphonomethoxy)-propyl]-2,6-diaminopurine	0.9	*In vitro*	[98]
		HPMPO-DAPy	(*R*)-(2,4-diamino-3-hydroxy-6-[2-(phosphono-methoxy)propoxy])- pyrimidine	5.1	*In vitro*	[98]
	PMEderivatives	PMEA	Adefovir	18–44	*In vitro*	[72,73,75,98]
		PMEDAP	(9-[2-(phosphonomethoxy)ethyl]-2,6-diamino-purine	16	*In vitro*	[98]
		PMEO-DAPy	2,4-diamino-6-[2-(phosphono-methoxy)ethoxy]-pyrimidine	12	*In vitro*	[98]
	PMPderivatives	PMPA	Tenofovir	>150	*In vitro*	Our unpublished data
Pyrophosphate analog		PFA	Foscarnet sodium	34–39	Cohort study	[35,36,74,75]
Non-nucleoside inhibitors	4-oxo-dihydroquinolines	183792, 529311, 568561, 570886		1.9–11.1 ^c^	*In vitro*	[99]
	Pyrimidoquinoline analog	NSC 373989	(5-((3-(dimethylamino)propyl)amino) -3,10-dimethy-lpyrimido[4,5-b] quinoline-2,4(3H,-10H)-dione)	1.9	*In vitro*	[100]

^a^ Concentration required to reduces KSHV DNA synthesis in TPA-stimulated BCBL-1 cells by 50% measured by real-time qPCR. The values are the range of the mean EC_50s_ of independent experiments as published in the original reports; ^b^ EC_50_ measured by flow cytometry; ^c^ EC_50_ measured by DNA hybridization assay. ^d^ Antiviral drug efficacy was evaluated *in vitro*, *in vivo* (MHV-68 mouse model) or in patients (cohort study, pilot study of randomized clinical trials). HPMP, 3-hydroxy-2-(phosphonomethoxy)propyl; PME, 2-(phosphonomethoxy)ethyl; PMP, 2-(phosphonomethoxy)propyl.

### 4.2. DNA Polymerase Inhibitors: Acyclic Nucleoside Phosphonates

The first ANP to be accredited with broad-spectrum antiviral activity against DNA viruses was (S)-9-(3-hydroxy-2-phosphonomethoxy-propyl)adenine or HPMPA [101]. In this nucleotide analog, the phosphate linkage (P-O-C) was replaced by the phosphonate (P-C-O) linkage, which was critical for the observed biological activity [102]. Subsequently, CDV was described as an antiviral agent active against HCMV and other DNA viruses [64]. In 1996, CDV was licensed for clinical use, under the trade name of Vistide^®^, for the treatment of HCMV retinitis in AIDS patients [68]. CDV is administered intravenously with the concomitant oral administration of probenecid, in order to block the drug uptake by the organic anion transporter in the proximal renal tubular cells that is responsible for the drug-related nephrotoxicity [102]. 

**Figure 3:**
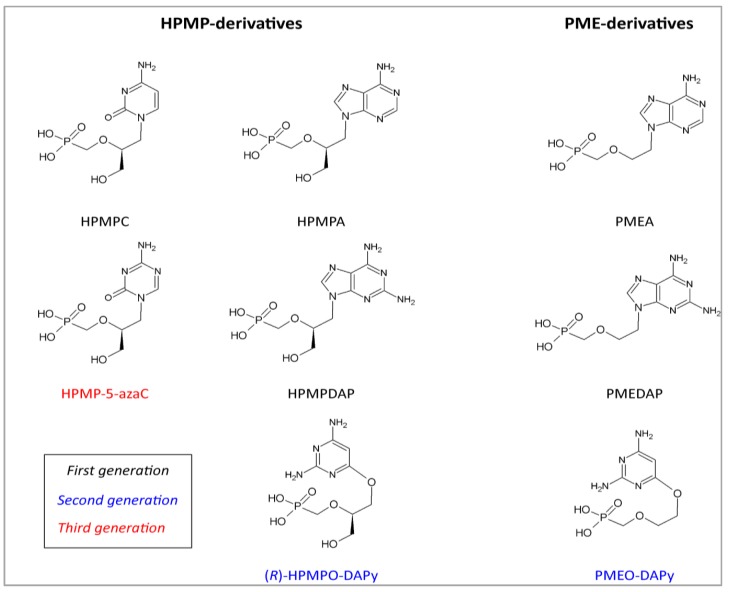
Structures of ANPs that exhibit anti-herpetic activity. Cidofovir (CDV, HPMPC), HPMPA and adefovir (PMEA) belong together with (*S*)-HPMPDAP and PMEDAP to the first generation of ANPs. (*R*)-HPMPO-DAPy and PMEO-DAPy belong to the second generation of ANPs, and HPMP-5-azaC is a molecule of the third generation of ANPs.

HPMPA and CDV (HPMPC) are ANPs classified as ‘HPMP’ (3-hydroxy-2-phosphonomethoxypropyl) derivatives, and differ from ‘PME’ (2-phosphonomethoxyethyl) derivatives, represented by adefovir (9-(2-phosphonomethoxyethyl) adenine, PMEA), according to their spectrum of antiviral activity (Figure 3) [62]. Adefovir was reported as an antiviral agent inhibiting *Herpesviridae*, *Hepadnaviridae*, and *Retroviridae* [68,101]. This drug was implemented for the treatment of chronic hepatitis B under the trade name of Hepsera^®^. PMPA or tenofovir is the representative of the ‘PMP’ (2-phosphonomethoxypropyl) derivatives of ANPs and has an antiviral spectrum restricted to *Hepadnaviridae* and *Retroviridae* [68]. The anti-HIV properties of tenofovir were first described in 1993 [101], and eight years later, the compound was licensed for clinical use for the treatment of HIV infections, under the trade name of Viread^®^ [103]. Meanwhile, tenofovir in its oral prodrug form, tenofovir disoproxil fumarate, has become one of the cornerstones for anti-HIV therapy [104]. Recently, Hepsera^®^ has been largely replaced by tenofovir, since the drug is approximately 30-times more potent against hepatitis B virus.

To accomplish their antiviral action, ANPs, must be first phosphorylated to their monophosphate form (MP) and, subsequently, to their diphosphate form (DP), which can be considered as the active metabolite that will finally interact with the viral DNA polymerase (Figure 2) [105]. These two phosphorylation steps are carried out by cellular enzymes. Of note, the intracellular phosphorylation of ANPs is thus independent of the herpesvirus-encoded TK or PK [68]. At the viral DNA polymerase level, the diphosphate form acts as a competitive inhibitor or alternate substrate with respect to the natural nucleoside, e.g., 2’-deoxycytidine-5’-triphosphate (dCTP) for CDV-DP, whereas the mechanism of action of adefovir is similar to that of CDV the integration of one molecule of adefovir at the 3’-end of the growing DNA chain terminates further chain elongation. On the other hand, CDV requires two consecutive (‘tandem’) incorporations to efficiently terminate DNA elongation by HCMV DNA polymerase [104].

As shown specifically for CDV, this compound offers a much longer antiviral response (several days) than nucleoside analogs, such as ACV, for which the antiviral response lasts for only a few hours [104,106] The prolonged antiviral action of CDV can be attributed to the long half-life of the CDV metabolites (CDV-MP, CDV-DP, and CDV phosphate-choline adduct) that are formed intracellular following uptake of CDV by the cells [107]. In particular the CDV phosphate-choline adduct serves as intracellular storage of CDV, since its intracellular half-life is 48 h [108].

A great deal of attention was given to the development of HPMPA analogs to improve its pharmacokinetic profile [109]. Promising anti-DNA virus effects were found for the 2,6-diaminopurine counterpart of HPMPA (*i.e.*, HPMPDAP) and PMEA (*i.e.*, PMEDAP) (Figure 3) [109]. The activity of HPMP-derivatives against DNA viruses is generally higher compared to their counterparts in the PME-series [110]. This was also seen against KSHV for which HPMP-derivatives were 10- to 100-fold more inhibitory than PME-derivatives (Table 2). 

A second generation of ANPs has been described including the 6-[2-phosphono-methoxy)alkoxy]-2,4-diaminopyrimidines (DAPy). These compounds fall into two categories with as prototypes (*R*)-HPMPO-DAPy and PMEO-DAPy (Figure 3) [109]. The anti-DNA virus activity of (*R*)-HPMPO-DAPy is similar to that of CDV. Nevertheless, its inhibitory activity was five-fold less pronounced against KSHV than CDV (Table 2) [98]. 

A new class of promising antiviral compounds came with the discovery of ANPs bearing a triazine ring, especially 5-azacytosine as a base component [111]. The 5-azacytosine analog of CDV, *i.e.*, 1-(S)-[3-hydroxy-2-(phosphonomethoxy)propyl]-5-azacytosine (HPMP-5-azaC) (Figure 3), showed similar or higher anti-herpetic activity, including anti-KSHV activity, as compared to CDV. However, it remains to be investigated whether CDV derivatives, such as HPMP-5-azaC, would be more efficacious than CDV in the treatment of KSHV infections. 

In addition to the dose-dependent nephrotoxicity of CDV, another major disadvantage that has restrained its use is its low oral bioavailability, due to the presence of the phosphonate group [61]. In order to achieve better oral bioavailability, the phosphonate group of the drug can be transformed to a phosphonic ester or amidate, which is enzymatically cleaved to the parent drug after passing the intestinal barrier, or inside the cells [112]. A considerable number of ANP prodrugs have been evaluated, but only a few of them passed preclinical studies. Additionally, lipophilic esters constitute an important class of phosphate and phosphonate prodrugs [112]. 

Particularly, the hexadecyloxypropyl (HDP) prodrug of CDV or CMX001 (brincidofovir) has shown promising results and is currently being developed as prophylactic and preemptive therapy of viral DNA infections [113]. A pharmacokinetic and safety study in humans reported that oral administration of CMX001 at different doses are well tolerated, with no dose-limiting toxicity, particularly, no nephrotoxicity or myelotoxicity, which are the dose-limiting toxicities of CDV or GCV, respectively [114]. In addition, CMX001 given orally at a dose of 100 mg to patients that received allogeneic hematopoietic-cell transplantation was shown to be well tolerated (*i.e.*, diarrhea was the only dose limiting adverse effect) and effective (*i.e.*, it reduced the incidence of HCMV events in these patients) [115]. In our studies, KSHV was similarly sensitive to CMX001 or CDV in BCBL-1 cells with EC_50_ values of 0.7 µM and 1.3 µM, respectively (Table 2) [98].

### 4.3. DNA Polymerase Inhibitors: Pyrophosphate Analogs

Phosphonoacetic acid and phosphonoformic acid are pyrophosphate analogs and non-competitive inhibitors of viral DNA polymerases by binding to the pyrophosphate-binding site of the enzyme. Hence, these compounds block the release of the pyrophosphate from the terminal nucleoside triphosphate added onto the growing DNA chain (Figure 2) [116]. PFA, the trisodium salt of phosphonoformic acid (foscarnet), is only available as an intravenous preparation and can cause nephrotoxicity and significant electrolyte disturbances [70]. PFA can be considered as a second-line therapy and its use is reserved to HSV, VZV, and HCMV patients that have failed ACV or GCV therapy due to viral resistance or that cannot be treated with GCV due to side effects of the drug [62]. PFA has been used as antiviral for the treatment of KSHV and, despite its lower activity against KSHV replication *in vitro* compared to GCV and CDV, this drug has shown efficacy in KS patients [35,36].

### 4.4. DNA Polymerase Inhibitors: Non-Nucleoside Analogs

The 4-oxo-dihydroquinolines derivatives have been reported to have activity against most herpesviruses, but not against other DNA or RNA viruses. They were found to inhibit the polymerases of HSV, VZV, CMV, EBV and KSHV *in vitro*, and were shown active against a variety of ACV-, GCV-, and PFA-resistant HSV and HCMV mutants [99]. By means of flow cytometry, the antiviral activity (EC50 values) of four 4-oxo-dihydroquinolines against KSHV were calculated and ranged from 1.9 µM to 11 µM (Table 2) [99].

### 4.5. Non-DNA Polymerase Inhibitors that Target Viral DNA Synthesis

KSHV encodes for its own DNA polymerase processivity factor, which is required for lytic viral replication and allows the viral DNA polymerase to synthesize extended stretches of DNA without dissociating from the template [117]. The highly specific interaction between the polymerase and the processivity factor may be effectively targeted by small molecules to inhibit (i) the enzymatic activity of the polymerase, (ii) the interaction between the two proteins or (iii) the function of the processivity factor itself. Dorjsuren and colleagues employed an *in vitro* assay to screen compounds inhibiting KSHV DNA synthesis through targeting the viral DNA polymerase/processivity factor complex [100]. Of 18 active compounds examined, NSC 373989 was shown to specifically block lytic KSHV DNA replication in phorbol-12-myristate-13-acetate (PMA)-stimulated KSHV-infected BCBL-1 cells (Table 2) [100]. The active compounds have structures similar to various classes of topoisomerase II inhibitors, and these results suggested that certain agents may serve as dual inhibitors of human DNA topoisomerase II as well as of KSHV DNA synthesis [100,117]. For example, (+)-Rutamarin, a topoisomerase II inhibitor isolated from plants, such as Ruta graveolens L, was also found to selectively inhibit KSHV replication [118].

### 4.6. Other Inhibitors of KSHV Replication

A number of compounds purified from plants are known to inhibit KSHV replication. For instance, angelicin, isolated from the seeds of *Psoralea corylifolia*, is able to inhibit lytic replication of γ-herpesviruses during the early stage of *de novo* infection and/or reactivation [119]. Other drugs inhibit the immediately-early Rta promoter of KSHV, or alter the interaction of cellular transcription factors with Rta. They include resveratrol (a non-flavonoid polyphenol present in *Polygonum cuspidatum*) and the major cannabinoid compound of marijuana, delta-9 tetrahydrocannabinol [120,121]. 

Zhang and colleagues have demonstrated that inhibition of KSHV replication could be achieved by the use of phosphorodiamidate morpholin oligomers (PMO) which are short single-stranded DNA oligomers with a modified backbone conferring resistance to nucleases [122]. In this study, Rta (KSHV replication and transcription activator) and LANA (latency-associated nuclear antigen) mRNAs were targeted by antisense peptide-conjugated PMO in PEL cells, resulting is a significant decrease in viral DNA levels as well as in the expression of several KSHV early and late genes.

Herpesviruses express a structurally and functionally conserved dimeric protease required for capsid assembly during lytic replication. Herpesvirus proteases do not resemble to any known protease fold pattern and are thus classified into a separate family of serine proteases [123]. Initial attempts to inhibit herpesvirus proteases targeted the active site of the enzyme, relying heavily on chemical structures for covalent inhibition and/or peptidomimetic scaffolds. Specifically targeting the active site of herpesvirus proteases have not yet result in pharmacologically viable lead compounds despite some *in vitro* success [124,125,126]. Craik’s group reported on a small molecule, DD2 (a benzyl-substituted 4-(pyridine-2-amido) benzoic acid), able to disrupt dimerization of KSHV protease by trapping an inactive monomeric conformation [127,128]. Two DD2 analogues generated through carboxylate biosteric replacement were shown to inhibit proteases of all three herpesvirus subfamilies (*i.e.*, α, β, and γ herpesvirinae) [129]. 

### 4.7. Potential Novel Drug Targets in KSHV

Nucleoside analogs are the leading compounds for treating or suppressing herpesvirus infections for more than 50 years now. ACV, PCV and their orally bioavailable prodrugs may not be fully effective, but they have been remarkably free from any toxic side-effects. While it will be very difficult for new compounds to match these favorable properties, not only novel potential viral targets are being explored as therapy for herpesvirus infections, including immediately-early viral proteins, the viral helicase-primase and the viral terminase, but also cellular proteins that are important for viral replication. While several studies have provided proof-of-principle that helicase-primase and terminase inhibitors can be effective antiviral against α- and β-herpesviruses in cell culture and in humans [130,131,132], these new viral targets have not yet been explored for γ-herpesviruses, such as KSHV. 

## 5. Cellular Targets

There is an abundance of evidence that host cell protein kinases, and the downstream pathways that they control, play a critical role in herpesvirus infection [133]. Inhibitors that target these host proteins might act as antiviral agents, yet, the risk for cytotoxicity and side effects increases by targeting host protein kinases [134]. Suppression of virus replication by a number of small-molecule inhibitors of cellular protein kinases has been demonstrated *in vitro* and several inhibitors have been incorporated into clinical trials examining their efficacies for the treatment of cancers [133]. Cellular serine/threonine protein kinases, which play an important role during the course of a KSHV infection, are mTOR, cyclic-dependent kinases, casein kinase 2, p90 ribosomal S6 kinases and PI3K, as well as tyrosine kinases, such as vascular-endothelial growth factor receptor, ephrin A2 and platelet-derived growth factor receptors [133]. Rapamycin, an mTOR inhibitor, was shown to prevent and induce regression of KS by inhibiting the expression of immediately-early proteins (Zta and Rta) of KSHV [135]. Dasatinib, an ATP-competitive tyrosine kinase inhibitor that inhibits multiple tyrosine kinases including EphA2, significantly reduces KSHV infection when cells are pretreated [133]. Because ephrin A2 functions as a cellular receptor for KSHV infection of endothelial cells, inhibitors of this tyrosine kinase may show promise as anti-KSHV agents.

## 6. Animal Models for Antiviral Efficacy Evaluation

MHV-68 infection in immunocompetent (BALB/c) mice has been well studied as an animal model for addressing fundamental aspects of KSHV pathogenesis and/or immunity [136,137,138]. This mouse model proved to be adequate for vaccination studies and for the investigation of strategies that modulate the tumorigenicity of virus-infected cells [139]. 

Infection of mice with murine γ-herpesvirus 68 (MHV-68) has been exploited as an experimental model to explore proof-of-principle vaccination strategies, such as MHV-68 subunit vaccines targeting lytic and latency-associated viral proteins, heat-inactivated MHV-68 virions and MHV-68 replication-deficient viruses. These vaccines were able to reduce the level of MHV-68 acute infection, but had little impact on long-term latency establishment [140,141,142,143,144,145,146,147,148,149,150,151]. Developing a therapeutic vaccine to increase the immune control of KSHV lytic replication and to decrease the KSHV viral load in people already infected may reduce the risk of KS and even virus shedding and transmission [152]. Since disease incidence in the majority of KSHV-infected people is low, scientific interests and efforts to develop a KSHV vaccine have been limited. Another major obstacle is the lack of an amenable animal model to evaluate the protective effects [153]. However, the access to a KSHV vaccine would have an impact on people that are at high risk of developing tumors, such as in HIV patients, immunosuppressed individuals, or for persons living in endemic African areas [153]. 

Additionally, the MHV-68 mouse model is particularly useful for the evaluation of the efficacy of antiviral agents that target the viral lytic cycle, since viral replication occurs in the lung of infected mice. Previously, distinct endpoints have been used to evaluate the efficacy of antiviral agents *in vivo*, such as mortality in MHV-68 immunocompromised mice [93] or inhibition of viral replication in lungs of immunocompetent mice [154]. 

Different ways have been used to set up KSHV infection in mice. In one model, purified virus is injected intravenously to NOD/SCID mice with severe combined immunodeficiency affecting T- and B-lymphocyte development as well as with Natural Killer (NK) cell, macrophage and granulocyte numbers and function reduced [155]. This model is suited to evaluate longitudinal patterns of viral gene expression, cell tropism and immune responses. Some NOD/SCID mice implanted with functional human hematopoietic tissue grafts (NOD/SCID-hu) were shown to produce human KSHV‑specific antibodies [155]. Furthermore, GCV treatment of these chimeric mice at the time of inoculation led to prolonged but reversible suppression of viral DNA and RNA levels. A second model used NOD/SCID mice reconstituted with KSHV-infected CD34^+^ hematopoietic progenitor cells (HPC) where it was shown that the virus establishes persistent infection in NOD/SCID mice and disseminated following differentiation of infected HPCs into the B-cell and monocytes linkages [156]. 

Growth of PEL derived cells lines as xenografts in immune deficient mice has been used to study the *in vivo* effects of therapeutic strategies for KSHV-associated malignancies [157,158,159,160]. A concern of this xenograft model is whether they entirely reflect clinical presentations of KSHV PEL. A recent study using a PEL xenograft model by intraperitoneal injection of KSHV PEL cells into the peritoneal cavity of NOD/SCID mice found that these animals not only developed massive ascites but also single or multiple solid tumors on various tissues in ~70%–80% of animals. Although this xenograft model can be used for the study of effusion and solid lymphoma observed in patients, tumor cells grown *in vivo* displayed unique features (including viral lytic gene expression profile, rate of solid tumor development and tumor microenvironment) to those passed *in vitro* [161].

## 7. Conclusions

Since the discovery of KSHV 20 years ago, little progress has been made towards therapies directed against this oncogenic virus. In the absence of molecules targeting viral latency and FDA-approved antiviral agents for the treatment of KSHV infections, few compounds have been evaluated, mostly *in vitro*, and all targeting the viral DNA polymerase. Previously, GCV was shown to be the most effective drug among the marketed antiviral agents for the treatment of KS patients. In contrast to GCV, other PK-dependent drugs, such as ACV, PCV, H2G and A-5021, showed weak or no anti‑KSHV activity, while TK-dependent drugs, such as HDVD, may be suitable drug candidates. Most importantly, because of the unclear role of antiviral therapies targeting lytic phase in the prevention or treatment of KSHV-induced diseases, randomized controlled clinical trials are needed to determine their true efficacy in different clinical settings. Furthermore, it would be of interest to investigate the role and potential of novel ANPs in the treatment of KSHV infections, since CDV therapy has shown unclear outcomes in patients. Finally, more efforts should be invested to examine the potential of non-nucleoside inhibitors against KSHV replication, as well as drugs that target viral proteins other than the viral DNA polymerase, since this proof-of-principle has been shown beneficial for other herpesviruses, such as HSV and HCMV. 
